# The long-term consequences of the hypospadias salvage repair issue

**DOI:** 10.1186/s12887-024-04534-3

**Published:** 2024-01-19

**Authors:** Hisham M. Hammouda, Ahmed A. Shahat, Ahmed S. Safwat, Taha M. Taha

**Affiliations:** Pediatric Urology Division, Urology Department, Assiut University, Assiut, Egypt

**Keywords:** Failed hypospadias-repair-long term

## Abstract

**Purpose:**

To present the long-term results of redo-hypospadias at our tertiary referral center following a failed prior repair.

**Methods:**

One hundred sixty-four individuals with a history of unsuccessful repairs qualified for our retrospective cohort study. Our inclusion criteria were as follows: pre-operative data that was accessible, redo-hypospadias that was successfully repaired, and at least three years of follow-up at the last hospital visit.

**Results:**

The mean patient age was 91.3 ± 21.1 months. The mean follow-up after successful repair was 41.3 ± 3.1 months. Ninety-two (group A) had one prior repair, and 72 (group B) had 2 or 3 repairs. Group A underwent six primary techniques: 32 underwent Onlay Island Flap (OIF), 10 underwent Mathieu, 12 underwent Tubularized Incised Plate Urethroplasty (TIPU), 8 underwent Urethral Mobilization (UM), and 34 underwent Buccal Mucosal Graft (BMG) { dorsal inlay Graft Urethroplasty (DIGU) in 4 and staged BMG in 30 patients}. In group B, four procedures were used: TIPU in 4, UM in 6, and BMG in 62 (staged BMG in 50 cases and DIGU in 12).

**Conclusions:**

The selected type of repair will depend on many factors, like residual healthy local skin and expertise. Safe techniques for repair of redo hypospadias after its 1st failure include TIPU, Mathieu, UM, OIF, and DIGU for distal varieties. After 2nd or 3rd repair DIGU, UM, and TIPU can be performed in distal types, while staged BMG can be applied for proximal ones.

## Introduction

Treatment of patients with failed (redo) hypospadias repairs is a challenging procedure. The need for a salvage radical approach in the presence of significant scarring and deficient genital skin leaves the surgeon with less than ideal circumstances. Different techniques have been reported in our study aimed at addressing a good approach to repair. The surgeon (technique selection and surgeon expertise) and the procedure are very important issues [1]. Repairs performed by an experienced pediatric urologist were associated with improved outcomes that could only be evaluated by long-term studies [2]. We tried to conduct long-term experience in the management of failed hypospadias repairs in a single referral center. Also, we postulated a logarithm or road map for solving this dilemma.

## Materials and methods

According to our retrospective hospital records, 244 patients had redo-repair of failed hypospadias in the form of combined glans and urethral dehiscence or urethral stricture between January 2000 and January 2018; however, 164 of those patients were eligible for inclusion in our retrospective cohort study. A functional urethra free of strictures, diverticulum, or recurrent fistulas, along with a slit like meatus at the summit of conical shaped glans, is what we meant by a consecutive outcome. Our inclusion criteria included having received documented legal consent and ethical permission by our Institutional Review Board, in addition to having had the hypopadias successfully repaired for three years or more since the last follow-up visit and having access to pre-operative data. Informed consent was obtained from all the parents of participants.

We excluded patients who are waiting for repair of any complications or not available for follow up. Our data charts included: age at repair, operative data of previous approaches and complications, thorough examination of penis and scrotum, satisfaction level of both children and parents.

Statistical analysis: Data analysis was performed via Microsoft Excel 2019. Quantative data were processed as mean ± standard deviation (SD), while qualitative data were expressed as numbers and percentages.

## Results

**Fig. 1:**
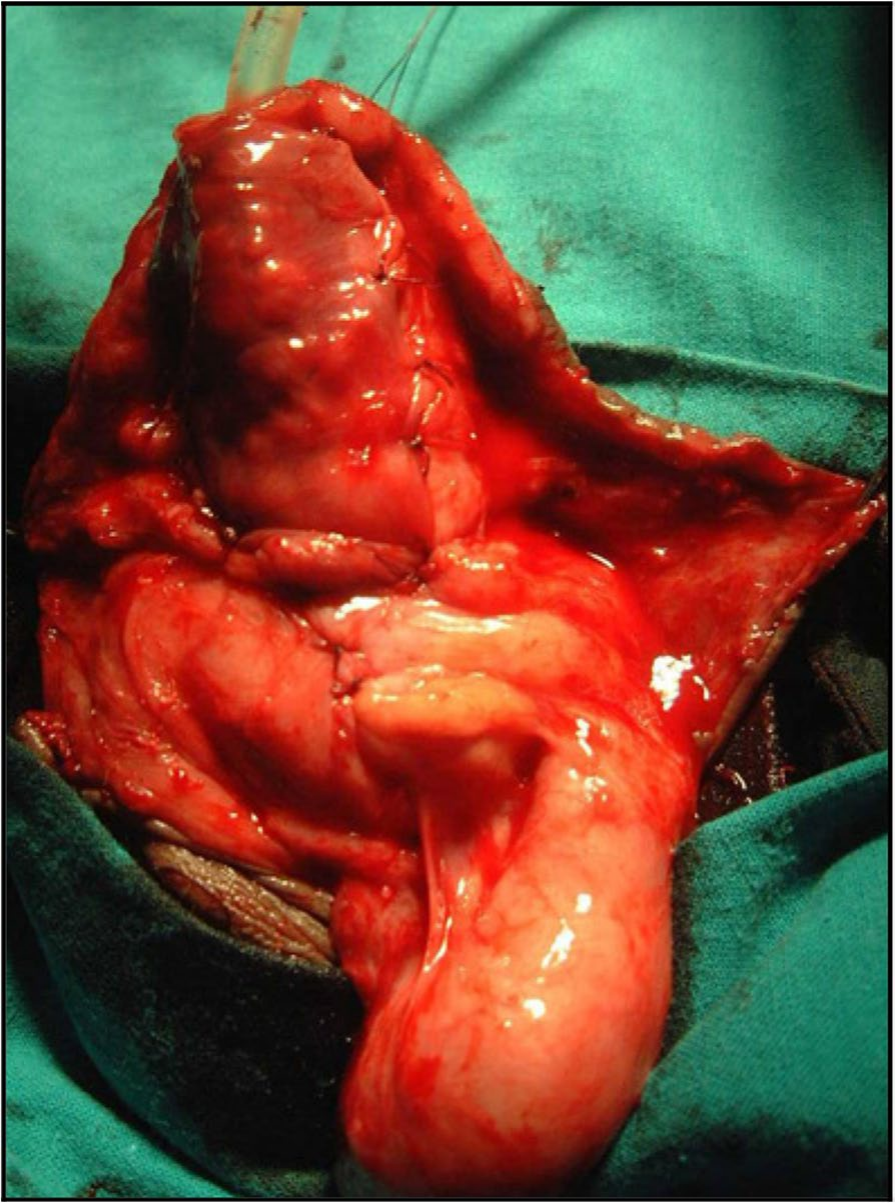
2nd layer tunica vaginalis cover

**Fig. 2:**
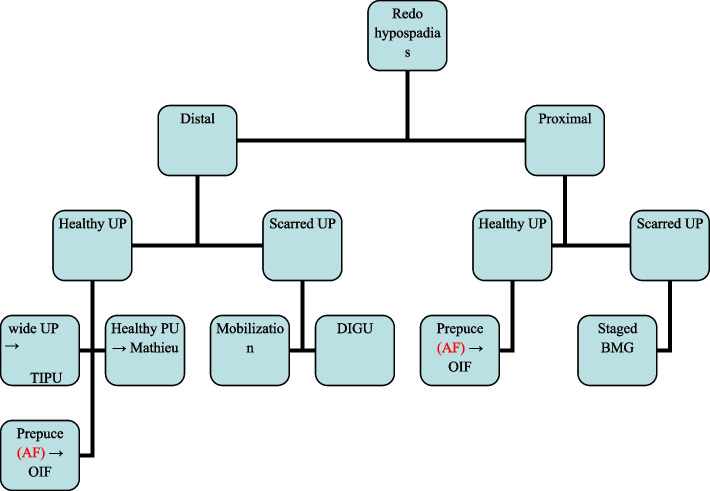
Logarithm for repair of redohypospadias

Mean patient age was 91.3 ± 21.1 months. Six primary techniques were applied, namely:, Onlay island flap (OIF) [3], Mathieu [4], and tubularized incised plate urethroplasty (TIPU) [5], urethral mobilization (UM) [6] and buccal mucosa graft (BMG) [7] from either the lower lip or inner cheek. BMG was done as one stage DIGU [8] in case of distal hypospadiac opening or staged in presence of proximal variety [9]. Multiple layers covering of urethral suture line were included as crucial step in all repairs (Fig. 1).Urethral urinary diversion was performed via silicone feeding tube. The mean follow-up after successful repair was 41.3 ± 3.1 months. Both Table 1 and Fig. 2 illustrate types and our logarithm for operations used for redo-hypospadias repair respectively. Ninety-two ( 56.1%) had single previous repair (group A), 42 (25.6%) had 2 previous repairs and 30 (18.3%) had 3 repairs (group B). All previous failed repairs had resulted in a meatus in an unacceptable position. Classification of the failed cases according to the site of meatus into distal (which includes coronal and distal) which was recorded in 38 (41.3%) in group A and 22 (30.6%) in group B, and proximal (which includes mid penile, proximal penile and peno-scrotal) was reported in 54 (58.7%) in group A and 50 (69.4%) in group B. In group A, OIF was performed in 28 (4 distal after failed Mathieu) and in 24 proximal after failed TIPU), Mathieu was performed in 10 distal (after failed TIPU), TIPU was performed in 12 distal (after failed Mathieu in 8 and failed OIF in 4), UM was performed in 8 distal (after failed 6 TIPU and 2 Mathieu), dorsal inlay graft urethroplasty (DIGU) using BMG in 4 distal (after failed TIPU) and staged BMG in 30 proximal patients (14 after TIPU, 10 after TPIT (transverse preputial island tube), and 6 after OIF).

**Table 1:** Types of successful operations used for redo-hypospadias repair

Total number (164)		Group A (92)		Group B (72)		
Operation		Distal (38)	Proximal (54)	Distal (22)	Proximal (50)	
-OIF		4	24	-	-	
-Mathieu		10	-	-	-	
-TIPU		12	-	4	-	
-UM		8	-	6	-	
- DIGU		4	-	12		
- Staged BMG		-	30	-	50	
		OIF	TIPU	Mathieu	TPIF	BMG
Distal						
-TIPU	4	4	-	-		-
-UM	6	-	4	2		-
-DIGU	12	2	6	4		6
Proximal						
-BMG	50	6	30		14	50

**Fig. 3:**
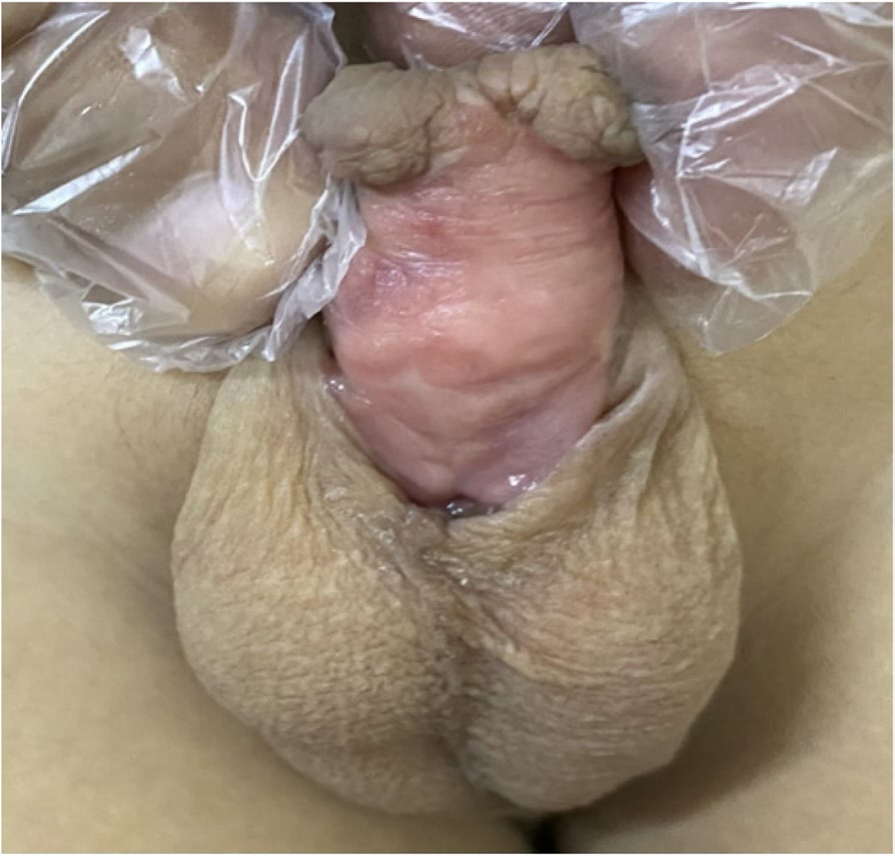
Intra-operative 1st. stage BMG

**Fig. 4:**
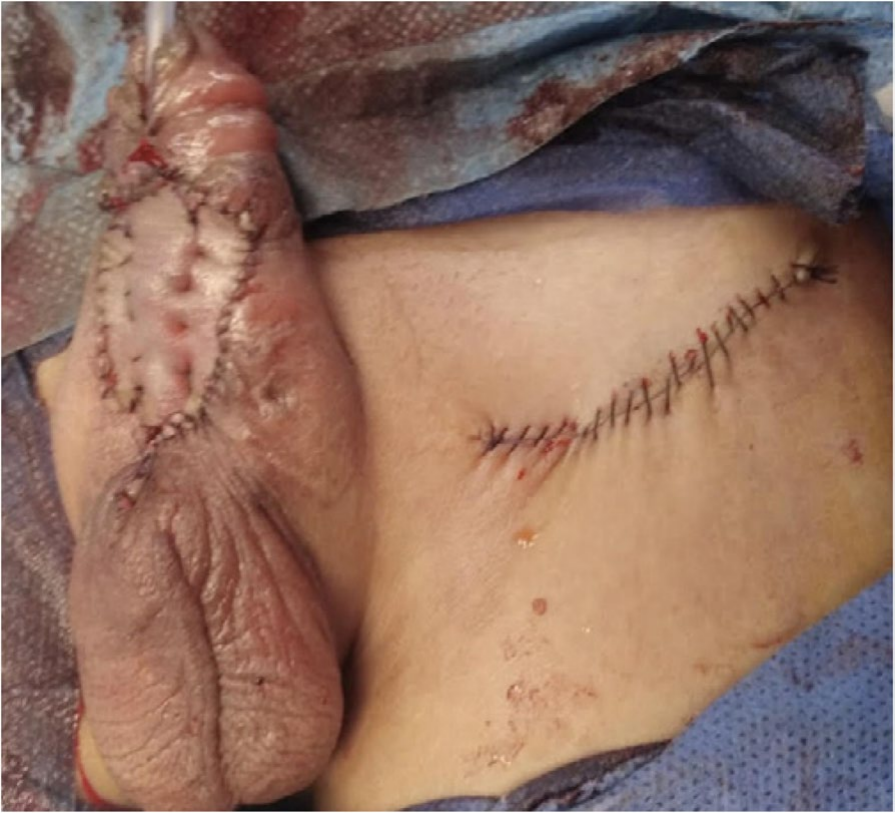
Penile cover by full thickness free skin graft

In group B, TIPU was done in 4 distal (after failed OIF), UM was done in 6 distal (after failed TIPU in 4 and Mathieu in 2), DIGU used BMG in 12 distal (after failed TIPU in 6 and Mathieu in 4 and OIF in 2), and staged BMG (Fig. 3) in 50 proximal (after failed TIPU in 30), TPIF in 14, and OIF in 6 cases. Deficient penile skin cover was reported in 8 proximal cases in group B and managed by full-thickness skin graft (Fig. 4).

**Fig. 5:**
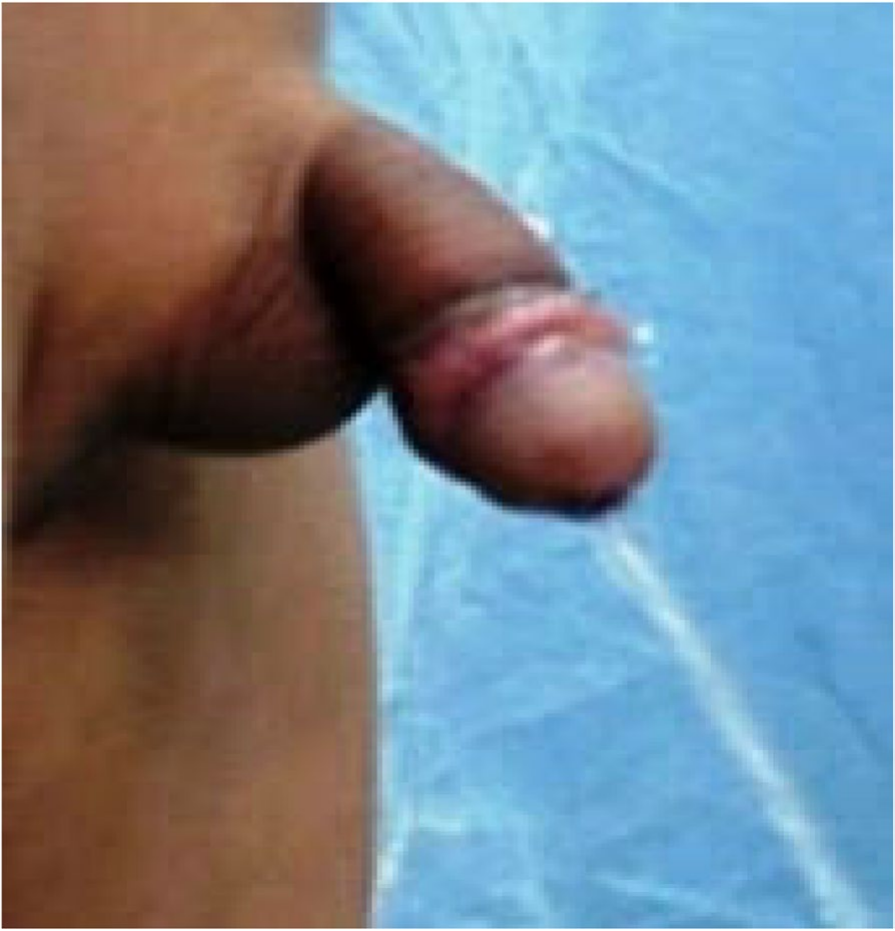
Satisfactory result of redo- hypospadias

The second stage of BMG was applied 6 months after the first stage (range 7.1 ± 1.2).Successful multilayer closure of small fistulas was recorded on 4/28, 2/10, 2/16, and 6/96 after OIF, Mathieu, TIPU, and BMG, respectively. There was no recorded meatal regression in 14 UM cases. A satisfactory response was achieved by both the child and his parents after successful hypospadias repair (Fig. 5).

## Discussion

A thorough assessment of the penis and scrotum is necessary in order to select the best plane and appropriate restoration techniques for redo-hypospadias. The strategy of repair will be the choice of residual healthy local skin, the excision of any scar tissue, and combinations of techniques. We do agree that available foreskin (AF) when a secondary salvage procedure for redo-hypospadias repair is needed offers a good chance for successful OIF repair [10]. Also, it's our belief, like others [11], that relocated penile skin during prior hypospadias repair can be used in a salvage pedicle flap procedure in the form of either a Mathieu urethroplasty or an island flap repair, which is only chosen from healthy, pliable skin. The Mathieu technique demonstrated good results in distal redo-hypospadias [12] and so OIF in both proximal and distal types [11]. TPIF does have a higher incidence of failed repair, and we have continued to extend our application of BMG as a salvage procedure after its failure [13]. In the mean time, TIPU reported favorable results in distal redo cases [14]. Also, TIPU is recommended if we have residual healthy urethral plate [15], grooved good-sized glans [16], and distal rather than proximal hypospadias [17, 18].

Technical issues during repair are fundamental demands for success, like the dissection of healthy skin flaps to preserve their vascular integrity and the multiple layers of hypospadias repair, especially by dartos and tunica vaginalis flaps [19, 20]. Urethral mobilization was reported as a successive salvage technique in distal redo-hypospadias [21], it is preferable to be used in older boys with a developed corpus spongiosum [6]. Its main drawback is meatal regression, which can be avoided if it is performed within its indications as previously described [6, 21], while it has the major advantage of avoiding any non-urethral tissue. We emphasize that salvage staged BMG is the cornerstone for repair of failed hypospadias in the absence of sufficient healthy penile skin, either in redo distal hypospadias as one-stage dorsal inlay graft [8] or in proximal type as staged procedure [9]. BMG can produce a well-vascularized urethral plate substitute; it reported a good success rate with good cosmetic outcomes in the management of redo hypospadias and its cripples. We agree that BMG is thinner and less bloody in the lower lip than in the inner cheek [22, 23]. However, BMG contracture is a known complication that was reported in 12% of redo cases that were successfully patched before. Deficient penile skin resurfacing represents an obstacle in some cases of redo-hypospadias repair. Numerous surgical techniques were described to get around that, like some incisions and suturing techniques, the use of tissue expanders, and free skin grafts [24]. We concur that a full-thickness skin graft is a wise choice [25]. The age of children in our cohort at surgery was similar to that of previous research [24, 25]. We claimed, like many investigators, that only long-term studies are capable of proper anatomical and functional evaluation of hypospadias repair [17, 26]., however, we do believe that satisfaction level by the child and his parents, together with good esthetic appearance, are extremely important after hypospadias repair [26].

Several researchers have argued that objective determination of functional and aesthetic post-hypospadias repair represents a forward step in its accurate evaluation; however, the approach is still controversial and non-standardized [17, 18]. Uroflowmetry was reported as a modality for functional evaluation of post-hypospadias repair with marked conflict about its timing, whether pre- or post-operatively, in addition to how to interpret the obstructed flow pattern in each type of repair [27]. Preoperative abnormal flowmetry in hypospadias could occur in a case of hypoplastic urethra with poor spongiosal cover, meatal stenosis, and associated bladder overactivity. Postoperative abnormal uroflow could be caused by a lack of spongy cover, meatal stenosis, fibrosis of stretched glans wings, and urethral stricture [28]. Aesthetic outcome and satisfaction level after hypospadias repair were postulated to be evaluated by different score systems [29, 30]. No any agreement between hypospadias patients and surgeons satisfaction with patient penile appearance was noted [29]. The Pediatric Penile Perception Score reported reliability and ease of use with certain prerequisites, with a strong suggestion that the assessment of the results by uninvolved urologists may diverge [30]. Multicenter collaborative long-term studies are requested for the establishment of surgical techniques for redo hypospadias repair, especially the crippled ones, and the detection of late neourethral stricture, residual penile curvature, and the growth of less androgen-sensitive non-genital skin urethroplasty.

## Conclusions

Repair of failed hypospadias depends on the availability of remaining tissues or the application of healthy tissue substitutes, together with surgical skills. TIPU, DIGU, UM, and Mathieu techniques are good options with good selection in cases of distal type; OIF is an excellent option to bridge any type if we have healthy prenuptial remnant or dorsal penile skin; and staged BMG is the master key for repair of proximal hypospadias cripples. Multiple layers of repair are mandatory. A full-thickness skin graft is an efficient maneuver for deficient penile skin cover. Repair of failed hypospadias is not for the occasional surgeon. We believe that long-term research can provide solid, reliable facts.

## Data Availability

The datasets generated and/or analyzed during the current study are available in the records of Assiut Urology Hospital, Assiut University.Also, it is available from the corresponding author on reasonable request.

## Abbreviations

BMG: Buccal mucosa graft
DIGU: Dorsal inlay graft urethroplasty
OIF: Onlay island flap (OIF)
TIPU: Tubularized incised plate urethroplasty
TPIT: Transverse preputial island tube
UM: Urethral mobilization
Fig.: Figure
UP: Urethral plate
PU: Proximal urethra
AP: Available fore skin
